# Impact of extreme physical exercise (28 consecutive marathons) on sleep time and structure

**DOI:** 10.1016/j.ijchp.2025.100586

**Published:** 2025-05-31

**Authors:** Gualberto Buela-Casal, Noelia Ruiz-Herrera, Alejandro Guillén-Riquelme, Carlos Zamarrón, Francisco Gude-Sampedro

**Affiliations:** a Sleep and Health Promotion Laboratory, Mind, Brain, and Behavior Research Center (CIMCYC), University of Granada, Spain; b International University of La Rioja, Spain; c Valencian International University, Spain; d University Hospital of Santiago de Compostela, Spain

**Keywords:** Sleep, Recovery, Athlete, PSG, Extreme physical exercise, Marathons

## Abstract

**Objective:**

It is known that physical exercise influences sleep, however, the effect in extreme sporting conditions has been less studied. We analyzed the sleep quality and duration of an athlete who ran 28 consecutive marathons (one per day).

**Methods:**

We evaluated with three polysomnography recordings to explore the sleep-dependent recovery process: Post-marathons, partial recovery, and baseline.

**Results:**

The participant reported a low sleep quality during the challenge, along with short sleep time, several awakenings, and daytime sleepiness. We observed differences in sleep duration, quality, and distribution in all variables evaluated through polysomnography. From baseline to the final condition after the physical test, we observed a progressive decrease in sleep time and sleep efficiency. In addition, we observed an increase in sleep onset and REM sleep latencies, a 45.8 % increase in slow wave sleep, which affects the complete sleep structure after physical exertion. REM decreased by up to 14.4 % because of intense and continuous physical exercise, and with respect to SWS (38.5 %) this represents an increase in SWS of 267 % with respect to REM.

**Conclusion:**

sleep is affected by high-intensity physical exercise and is modulated by the recovery process. The results of this study highlight the importance of SWS in the recovery from physical fatigue due to the effect of extreme physical exercise, which is demonstrated by the enormous increase in SWS that accounts for almost 40 % of the Total Sleep Time, surpassing even the stage 2 percentage. It is also shown that REM sleep has no role in the recovery from physical fatigue, as it is in fact considerably decreased by the effect of extreme physical exercise. Previous studies had not reported results with these magnitudes.

Numerous researchers have investigated the effects of sports on sleep because sleep is essential for physical recovery (Fox et al., 2020; Halson & Juliff, 2017; Marshall & Turner, 2016; Xie et al., 2013) and fundamental to achieve a proper training and successful performance (Chennaoui et al., 2015; Daniel et al., 2024; Juliff et al., 2015; Sargent et al., Roach, 2014). Sleep duration, sleep quality, and distribution of sleep stages are key factors that determine the restorative effect of sleep (Samuels, 2008) and have been shown to be modified by physical exercise because the body adjusts its sleep to the daily need for recovery (Driver & Taylor, 2000). For example, elite athletes seem to sleep less, have more fragmented sleep, and have poorer sleep efficiency than the general population (see Gupta et al., 2017, for a review; Juliff et al., 2015; Knufinke et al., 2018; Nikolaidis et al., 2023). In addition, sleep stage distribution is affected by exercise because slow-wave sleep (SWS) increases after periods of intense physical exercise (Halson & Juliff, 2017; Marshall & Turner, 2016) and REM sleep decreases in those cases (Driver & Taylor, 2000). Therefore, even though results generally have suggested that physical activity is beneficial for sleep (see Kredlow et al., 2015, for a review), it can be negatively affected by circumstances such as overtraining and stress (Botterill & Wilson, 2002).

Several studies have provided information about the sleep quality of athletes during competition through subjective and objective measures (Hausswirth et al., 2014; Kishi et al., 2024; Knufinke et al., 2018; Martin et al., 2018; Sargent et al., 2016; Tuomilehto et al., 2016). However, data on the post-exercise recovery process are still scarce. That is, few studies have focused on exploring (using objective measures) the degree to which objective sleep parameters return to baseline after high-intensity physical exercise in high-performance athletes.

Thus, this study had two main purposes: first, to examine the subjective sleep quality of an amateur athlete who ran 28 consecutive marathons (1 per day for 28 days, reaching 1217 km), and second, to evaluate the sleep recovery process through three polysomnographic (PSG) recordings (after the marathons [non-recovery condition], after 7 days of recovery [partial recovery], and after 21 days of recovery [baseline]).

## Methods

**Fig. 1:**
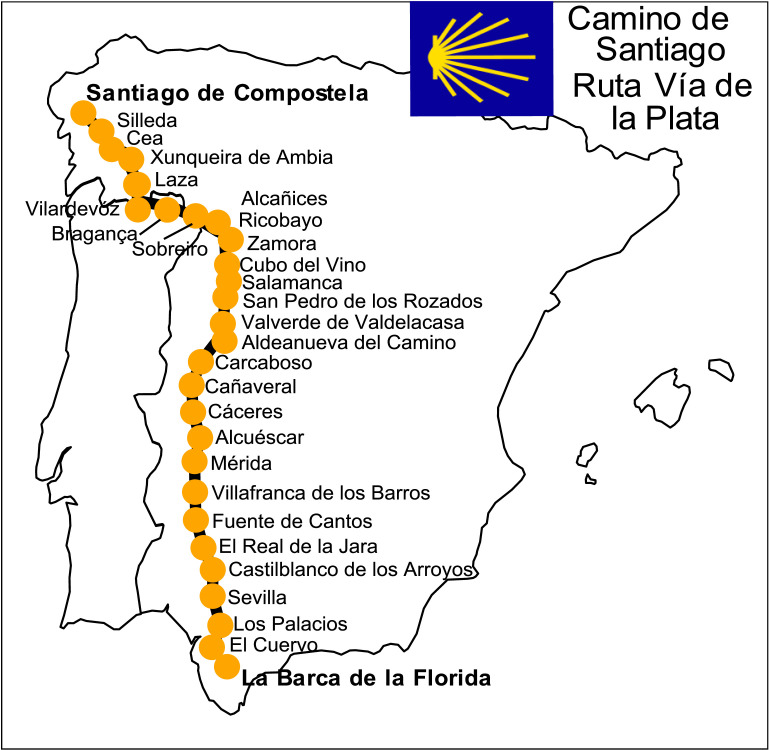
Diagram of the route followed and control points.

Because of the characteristics of the extraordinary physical effort exerted during the challenge, this was a single-case study. The athlete was a 51-year-old male from the province of Cadiz, Spain. His height is 182 cm, and his weight was 83 kg at the beginning of the challenge and 81 kg at the end of it, reaching 78 kg in the intermediate stages. The participant was not a professional athlete, so both the training and the marathons themselves were extraordinary circumstances in his usual sports routine. The 28 marathons took place along the Vía de la Plata route in Spain, covering 1217 km from Jerez - La Barca de la Florida (southern Spain) to Santiago de Compostela (northwestern Spain). The itinerary of the Ruta Vía de la Plata del Camino de Santiago was followed as much as possible to complete stages equivalent to a marathon. Specific details of each stage can be found in Fig. 1.

Each marathon was completed within a single day, covering an average distance of 43.5 km per stage.

Given the summer heat (September), the athlete typically started the runs early in the morning. The specific start times could vary slightly, but the start of the waking hours was always around 4 a.m.

### Sleep assessment

**Sleep diary.** The athlete filled out a sleep diary during the challenge in which he (and a nurse who was part of the support team) estimated the following parameters: time in bed, nocturnal sleep, naps, total sleep time, number of awakenings and their duration, perceived sleepiness at wake-up time, and perceived sleep quality. These last two parameters were evaluated on a scale ranging from 1 to 5, indicating the presence of lower to higher levels of the variables (e.g. 1 = low perceived sleepiness/sleep quality and 5 = high perceive sleepiness/sleep quality). In addition, the participant was asked about medication and other stimulant substances consumed, such as tobacco, caffeine, and alcohol.

**Polysomnographic recordings.** Three PSG recordings were obtained using SomnoScreen® Plus (Somnomedics, Randersacker, Germany) according to accepted guidelines for evaluation and correction (Berry et al., 2014). The lights went out between 00:00 a.m. and 1:00 a.m., the participant’s usual schedule, and he was allowed to sleep until his spontaneous awakening. The signals were recorded as follows: electroencephalogram (seven channels: two frontals [F3 and F4], three centrals [C3, Cz, and C4], two parietals [P3 and P4], and A1/A2 as reference); electrooculogram; electromyogram of the submentalis muscle; electromyogram of the left and anterior tibial muscles; and electrocardiogram. Impedance was maintained < 10 KΩ. Sleep signals were sampled at 256 Hz, and analyses were carried out with DOMINO light version 10.04. The participant’s sleep breathing pattern was recorded through nasal airflow thermistors and an abdominal and thoracic respiratory effort calibrator. Oxygen saturation was also assessed. Epochs containing technical artifacts or extremely high muscle activity causing saturation of amplifiers were carefully detected and marked for exclusion from the analysis. The participant was not allowed to consume stimulants substances such as caffeine or narcotics in the afternoon prior to PSG recordings.

### Procedure

The project was approved by the ethics committee of the University of Granada. After we obtained informed consent for participation and diffusion of results, we conducted the interview. The sleep diary was completed during each of the 28 nights of marathons. The first PSG recording was carried out at the University Hospital of Santiago de Compostela, performed immediately after the last stage (post-marathons) was completed. Because of the special characteristics of the study, an adaptive sleep assessment could not be conducted to eliminate the first night effect. We carried out the evaluation of partial recovery 1 week later and conducted the baseline assessment 21 days after the end of the challenge.

The partial recovery and baseline PSG recordings were carried out in the Sleep and Health Promotion Laboratory, at Universisty of Granada. In both centers, the PSG recording was performed using the same device, respecting the same electrode layout, using the same additional sensors, and analyzing the signal for the reports using the same software.

### Design

An inverse pre-post design was used. We performed the baseline sleep assessment 21 days after the completion of the challenge, with no high-intensity physical activity during that period. This design was implemented for several reasons: First, it ensured that the recovery observed in sleep parameters was not influenced by the pre-challenge training, as the participant had been training in high-altitude conditions (2320 m above sea level), which could have affected his sleep patterns. By waiting 21 days, we minimized any residual effects from the previous training. Second, the 21-day period allowed for sufficient time for partial physiological recovery, helping to stabilize the sleep state before the baseline assessment.

## Results

### Sleep diary

Sleep diary data can be found in Table 1. The athlete spent an average of 341.70 min in bed, not exceeding 400 min of sleep any night. He took daytime naps in 24 stages of the challenge, and in no case did the average nap time exceed 60 min. Perceived sleepiness after awakening was never below 2, and perceived sleep quality was above 3 on five occasions, with the highest quality observed only one night.

**Table 1:** Athlete's sleep diary data obtained during marathons.

Day	TIB	Nocturnal sleep	Daytime nap	TST	N° of awak.	Dur. of awak.	Sleepiness	Sleep quality
0^b^	180	154	0	154	3	22.5	5	2
1	365	340	0	340	1	5	3	3
2	285	245	0	245	0		4	3
3	295	257	30	287	1	3	3	3
4	237	171	40	211	2	11	2	2
5	284	249	40	289	1	5	4	3
6	390	305	0	305	2	5	3	4
7	325	295	50	345	0		2	2
8	270	229	30	259	2	12.5	3	3
9	295	260	20	280	1		3	2
10	375	315	40	355	1	10	4	3
11	330	275	30	305	2	10	3	3
12	405	360	30	390	1	5	3	4
13	390	275	40	315	2	10	2	2
14	340	225	40	265	2	15	3	3
15	330	135	30	165	2	5	2	2
16	330	145	50	195	3	10	2	3
17	420	260	30	290	1	5	4	4
18	390	230	40	270	2	10	2	2
19	320	240	50	290	2	10	3	3
20	375	270	60	330	1	5	2	3
21	285	270	30	300	2	5	3	2
22	360	315	40	355	2	10	3	3
23	345	310	30	340	1	5	3	4
24	375	265	50	315	2	10	4	5
25	375	205	40	245	2	15	2	3
26	360	255	50	305	3	5	2	2
27	375	280	60	340	2	5	2	2
28^a^								
29^b^	465	260	0	260	2	10	3	2
Mean (*S.D.*)	341.70 (46.12)	258.55 (52.94)	35.18 (46.12)	293.74 (51.61)	2 (0.74)	7.98 (3.51)	2.81 (0.73)	2.89 (0.80)

*Note.* TIB = time in bed; TST = total sleep time; TIB, nocturnal sleep, daytime nap, TST, and duration of awakenings are expressed in minutes. Perceived sleepiness and perceived sleep quality were evaluated on a scale ranging from 1 to 5.

a No sleep diary data were obtained for the night of challenge completion.

b Pre and post night data were not taken into account for the analysis.

### Polysomnographic recordings

Results of objective sleep measures in the three PSG recordings can be found in Table 2. Both time in bed and total sleep time were longer in the baseline condition compared to the other two, the shorter being that of the non-recovery condition. A remarkable progressive decrease in sleep efficiency was also observed in the non-recovery condition (68.1 %) with respect to partial recovery (83 %) and baseline (96.1 %). The sleep latency was considerably longer in the non-recovery condition than in the other two, with no noticeable differences between the latter two. Regarding sleep stage distribution, there was a progressive increase in REM latency from baseline (40.5 %) to the non-recovery condition (70.5 %), and we observed a decrease in REM sleep in non-recovery condition compared to the other two conditions. We observed a similar amount of Stage 2 sleep in the non-recovery and the partial recovery conditions, although it was less than at baseline. An increase of SWS in the post-marathons condition (38.5 % of total sleep time) with respect to partial recovery (26.9 %) and baseline (26.4 %) was observed.

**Table 2:** Objective sleep variables obtained for each condition.

	Baseline (night 21 post-marathon; complete recovery)	Partial recovery (night 7 post challenge)	Non-recovery (night 1 post challenge)
Time in bed	501	487.03	463.03
Total sleep time	482	404.00	315.50
Sleep efficiency ( %)	96.10	83.00	68.10
Sleep latency	0.73	3.73	92.55
Sleep period time	500	481.50	364.00
REM latency	40.50	50.50	70.50
REM ( %)	21.30	30.90	14.40
Stage 1( %)	8.30	5.90	11.60
Stage 2( %)	43.60	36.30	35.50
SWS( %)	26.70	26.90	38.50

*Note.* Data not indicated with (%) are expressed in minutes.

## Discussion

### Subjective sleep measures during the challenge

Despite the limitations associated with subjective techniques, the duration and quality of sleep perceived by the participant was low during the challenge, which is consistent with previous scientific literature in this respect (see Gupta et al., 2017, for a review; Hausswirth et al., 2014; Thornton et al., 2017). It is likely that the amount and quality of sleep perceived by the athlete were affected by sleeping in different environments and by the perceived stress (Fortunato & Harsh, 2006; Jackson & Gaston, 2019).

According to the challenge characteristics and the commitments of the participant with the organizing institutions, an ad libitum sleep schedule was not possible, and sleep quality was considerably below the recommended levels (Consensus Conference Panel et al., 2015). In addition, the number of awakenings reported by the participant provides evidence of the previously reported sleep fragmentation, along with the medium-high perceived sleepiness after awakening (see Gupta et al., 2017, for a review; Knufinke et al., 2018; Lastella et al., 2014).

### Polysomnographic measurement during the recovery process

The sleep time and quality of the participant after finishing the challenge was below normal according to recommendations (Hirshkowitz et al., 2015), which is in agreement with previous studies that reported a decrement in these variables in athletes with respect to controls (Hausswirth et al., 2014; Lastella et al., 2014). Previous studies using PSG recordings have revealed that sleep time decreased as the competition progressed (Léger et al., 2008). In this study, the amount of sleep of the participant increased progressively 7 and 21 days after the completion of the challenge. Furthermore, sleep efficiency was not adequate in either the non-recovery condition or the partial recovery condition (< 85 %; Ohayon et al., 2017), which is in accordance with scientific literature in which sleep efficiency is compromised in overreached athletes (Wall et al., 2003). This confirms that prolonged periods of extreme exercise intensity may worsen sleep duration and quality (Driver &Taylor, 2000) and supports the progressive changes of sleep during the recovery process.

Regarding sleep latency, we observed an increase in the non-recovery condition, that is, when training load was more extreme, supporting previous results in which sleep onset latencies were affected by exercise (Leeder et al., 2012). However, sleep latency was immediately favored by the recovery process as the values returned to baseline levels from the partial recovery measurement.

With respect sleep distribution, we observed noticeable changes in REM latency and REM sleep across the three measurements. These results confirm previous reports by showing a progressive increase in REM latency from baseline to the non-recovery condition and a decrease of REM sleep in the same direction (Netzer et al., 2001; Youngstedt et al., 1997). Furthermore, the amount of Stage 2 sleep was not within healthy ranges in any recording (Ohayon et al., 2004), although it was very close to normal values at baseline. The percentage of Stage 2 sleep, although similar, was lower in the partial-recovery and non-recovery conditions, probably due to the greater presence of SWS. In fact, and in agreement with previous results (Halson & Juliff, 2017; Marshall & Turner, 2016; Shapiro et al., 1981), a greater amount of SWS was observed in the non-recovery condition, thus supporting the idea of its essential role in physical recovery.

We acknowledge that measurements of sleep prior to the challenge would have been useful, as they would have provided baseline data to assess the participant's sleep health and rule out any symptoms of sleep disorders before the challenge. Unfortunately, this information was not available, limiting our ability to determine whether any pre-existing sleep disturbances influenced the sleep recovery process during the study. However, the fact of being able to perform 28 consecutive marathons and the rapid recovery of normal sleep parameters are not compatible with any sleep disorder (Buela-Casal et al., 2024).

In conclusion, not only did we confirm low perceived sleep quality under overreaching conditions, but we observed a progressive sleep-dependent recovery process in sleep duration, quality, and distribution. However, measurements of sleep prior to the attempt would have been appropriate, and would have added evidence to the participant sleep health, and rule out any symptoms of disorder prior to the challenge.

### Clinical implications and perspectives

Our results clearly indicate that prolonged periods of extreme exercise intensity may worsen sleep duration and quality and support the progressive changes of sleep during the recovery process. As previously reported, lack of sleep has a negative impact not only on athletic performance but also on physical and mental health (Simpson et al., 2017; Sutton, 2014). In fact, it has been observed that professional athletes from various disciplines suffer more physical problems when they have sleep problems (Biggins et al., 2019). Because sleep can optimize athletes’ recovery and performance, and to avoid physical problems resulting from sleep issues, coaches should use strategies to maximize sleep duration and quality (Simpson et al., 2017) and promote the sleep-dependent recovery process by establishing adequate protocols (see Hotfiel et al., 2019; Kölling et al., 2019, for reviews). Moreover, it is important to acknowledge that while regular physical activity has well-established health benefits, extreme endurance exercise may have a plateauing effect or even lead to adverse health outcomes. Some studies have indicated that excessive training intensities could potentially result in detrimental effects on cardiovascular health, immune function, and musculoskeletal integrity (e.g., Franklin et al., 2022). Therefore, optimizing the balance between exercise intensity and recovery is essential to avoid these negative consequences while promoting athletic performance and overall well-being.

More research is needed to elucidate why sleep latency and SWS values returned more drastically to the baseline than the other variables. In addition, it would be interesting to explore the impact of high-intensity physical exercise on the sleep microstructure, as well as to investigate sleep-dependent recovery protocols that could be used to optimize the process in different sports, for both individual and team athletes. With regard to other variables that could influence sleep and recovery, the association between physical activity and stress management is already known (Föhr et al., 2017). In this case, it would have been interesting to evaluate both the objective and subjective stress levels of the participant during the challenge in order to better understand the effect it has on the sleep-dependent recovery process. Our findings highlight the importance of sleep quality and recovery in athletes under extreme physical stress. Given the progressive improvements in sleep observed during the recovery process, interventions designed to optimize sleep in athletes could play a critical role in reducing recovery times and enhancing performance. Sleep-dependent recovery protocols, such as strategic sleep timing and environment adjustments, may help athletes recover more efficiently and return to peak performance levels more rapidly after intense physical exertion. Therefore, implementing individualized sleep strategies that align with athletes' circadian rhythms and training schedules could be essential for improving both recovery and long-term performance outcomes.

## Declaration of competing interest

Authors declare the absence of any conflict of interest.
